# Inosine Pranobex Deserves Attention as a Potential Immunomodulator to Achieve Early Alteration of the COVID-19 Disease Course

**DOI:** 10.3390/v13112246

**Published:** 2021-11-09

**Authors:** Jiří Beran, Marián Špajdel, Jiří Slíva

**Affiliations:** 1 Department for Tropical, Travel Medicine and Immunization, Institute of Postgraduate Health Education, 100 05 Prague, Czech Republic; 2 Department of Psychology, Faculty of Philosophy and Arts, Trnava University, 918 43 Trnava, Slovakia; marian.spajdel@truni.sk; 3 Department of Pharmacology, Third Faculty of Medicine, Charles University, 100 00 Prague, Czech Republic; slivaj@seznam.cz

**Keywords:** inosine pranobex, methisoprinol, isoprinosine, COVID-19, SARS-CoV-2, lymphopenia, immunomodulation, drug repurposing

## Abstract

Since its licensing in 1971, the synthetic compound inosine pranobex has been effectively combating viral infections, including herpes zoster, varicella, measles, and infections caused by the herpes simplex virus, human papillomavirus, Epstein–Barr virus, cytomegalovirus, and respiratory viruses. With the emergence of SARS-CoV-2, new and existing drugs have been intensively evaluated for their potential as COVID-19 medication. Due to its potent immunomodulatory properties, inosine pranobex, an orally administered drug with pleiotropic effects, can, during early treatment, alter the course of the disease. We describe the action of inosine pranobex in the body and give an overview of existing evidence collected to support further efforts to study this drug in a rigorous clinical trial setup.

## 1. A COVID-19 Treatment Resilient to New Variants Is Needed

Acute viral infections often have few, if any, treatment options apart from vaccination. The effort to create a sufficiently large portfolio of anti-viral drugs and treatments protocols is, therefore, as important as the effort to prevent individuals from contracting the virus. Although vaccination is the gold standard for preventing the spread of viral infections and their progression in those already infected, vaccinations have limitations. New virus variants can escape the vaccine, and hence a vaccine’s effectiveness can deteriorate over time. This risk is catalyzed by high viral incidence [1]. Nevertheless, the strengthening of the vaccination campaign represents the most effective means to combat the SARS-CoV-2 infection and prevent severe manifestations of the virus [2].

Unfortunately, resistance to vaccination will remain in a certain percentage of the population [3,4], and the waning of vaccine protection will presumably lead to resumed susceptibility to the pathogen and its new forms. A considerable portion of the population cannot be vaccinated for medical reasons or simply because a vaccine is not licensed for certain age groups, e.g., children.

These rather general concerns proved applicable to the current unprecedented COVID-19 pandemic caused by the severe acute respiratory syndrome coronavirus 2 (SARS-CoV-2). Since the outbreak in the late autumn of 2019, the pandemic has been imposing a tremendous global burden in terms of morbidity, mortality, and economic impact, and it is far from being successfully controlled [5,6]. COVID-19 poses a serious threat to the elderly and those with underlying risk factors such as obesity, diabetes, and chronic lung or heart diseases. Numerous vaccines have been introduced since December 2020, and their effect has been significant in terms of reducing the incidence of both symptomatic and asymptomatic cases [7]. With the emergence of new variants of the virus such as the alpha (B.1.1.7), beta (B.1.351, B.1.351.2, B.1.351.3), delta (B.1.617.2, AY.1, AY.2, AY.3), and gamma (P.1, P.1.1, P.1.2) fears of resumed growth in incidence are valid. It seems that the vaccines can provide substantial protection from acquiring the disease and developing symptoms leading to hospitalizations [8]. This is particularly obvious in countries where relatively high vaccine coverage was achieved [9]. However, it seems that the virus is capable of producing a stream of new variants and that virus itself will most likely persist in humans due to limited vaccine coverage. It is also questionable whether any realistically achievable vaccine coverage would be sufficient to prevent transmission of the virus globally since the vaccine itself may not limit the viral load, as recent findings have shown [10]. Therefore, in addition to the effort to reach high vaccination coverage and maintain vaccine effectiveness, the search for drugs to treat COVID-19 or as adjuvant therapy or supplements remains a high priority [11,12].

Along with the above-mentioned strategies, it is necessary also to evaluate the current guidelines and strategies to minimize the virus spread and ensure universal personal preventive and protective measures [13].

The knowledge and understanding of the SARS-CoV-2 virus are evolving, in terms of its epidemiology, pathogenicity, and clinical manifestations, which ultimately map the strategic path, towards effective and safe treatment and production of reliable and potent vaccines [14].

All aspects of SARS-CoV-2 virus and COVID-19 disease in adults and among children were already described in many publications [14,15,16]. Clinical manifestation of COVID-19 remains unchanged during the whole epidemic. In one of the largest reviews of a total of 114 pediatric cases with COVID-19, the main clinical features were mild symptoms including fever (64%), cough (35%), and rhinorrhea (16%). The laboratory findings were lymphopenia (33%), elevated D-dimer (52%), and C-reactive protein (40%) levels. Ground-like opacities were common radiological findings (54%) and there were reported patients (15%) with the multisystem inflammatory syndrome in children. No deaths were reported [15].

Clinical manifestation among adults and the elderly is different from children clinical picture in terms of consequences. Eighty studies were included in the meta-analysis, including 61,742 patients with confirmed COVID-19 infection. The most common symptoms among COVID-19 infected patients were fever 87% and cough 68%. The laboratory analysis showed that thrombocytosis was present in 61%, CRP was elevated in 79%, and lymphopenia in 57.5%. The most common radiographic signs were bilateral involvement in 81%, consolidation in 73.5%, and ground-glass opacity in 73.5% of patients. Case fatality rate (CFR) in <15 year-olds was 0.6%, in >50 year-olds was 39.5%, and in all range groups it was 6% [16].

## 2. Drug Repurposing as a Pragmatic Approach

Repurposing of existing anti-viral agents deserves attention since it may yield a fast and economical solution to both developed and undeveloped countries.

Since 2020 there has been a substantial effort to repurpose anti-viral compounds with presumed anti-viral effects to treat COVID-19. Several review papers are available [17,18,19,20]. Given the extent of the pandemic and the associated probability of new variants arising in communities with high prevalence and poor vaccination coverage, efforts to identify drug candidates with robust effects and resilient to new variants deserve attention. Unlike drugs that directly affect vital functions of a virus, which are typically anticipated by computational drug repurposing efforts, drugs that nonspecifically enhance the host’s response can be an effective therapeutic strategy for combating a broad spectrum of viral variants. Here, the goal can be less ambitious than the eradication of the virus from the body; instead, the aim is to reduce viral load, overall tissue destruction caused by the virus and accelerate the recovery of patients. The infection will still take place, yet with less pronounced symptoms, but a harmful, inadequate immune response to an excess of virus antigens in the body might be avoided. Infection with SARS-CoV-2 triggers a hyperactive inflammatory response, releasing a large amount of pro-inflammatory cytokines. This event, referred to as a “cytokine storm”, has been reported with lung injury, multi-organ failure, immunothrombosis and unfavorable prognosis in severe COVID-19 in several studies [12,21,22,23,24].

### 2.1. Lymphopenia as Key Factor for COVID-19 Outcome

Any successful control of a viral infection involves the complex interplay between diverse cell types associated with both innate and adaptive immunity. Natural killer (NK) cells are a type of innate lymphoid cell that plays an important role in the first line of immune defense against any viral infection, including COVID-19. They constitute the primary rapid, innate immune attack on virus-infected cells [25].

Lymphopenia, a major immunological abnormality that in up to 96.1% occurs in severe COVID-19 patients, is strongly associated with mortality rate [26,27]. Zheng et al. and unpublished data by Zhang et al. indicate lymphopenia was associated with mortality, particularly in patients with low blood proportions of CD3+ T, CD4+ T, and CD8+ T cells [28,29]. The proportion of blood lymphocytes has demonstrated the most significant and reliable correlation with disease progression in patients who died due to COVID-19. Hence, the percentage of blood lymphocytes can be considered as a valid and accurate indicator for the classification of COVID-19 patients in moderate, severe, and critical cases [30].

A small proportion of lymphocytes may express the main receptor for SARS-CoV-2, i.e., the angiotensin-converting enzyme 2 (ACE2). SARS-CoV-2 can also use ACE2-independent pathways to enter lymphocytes. Both SARS-CoV-2- and immune-mediated mechanisms may trigger lymphopenia by influencing lymphocyte production, survival, and tissue re-distribution. Metabolic and biochemical changes can also affect the production and survival of lymphocytes in COVID-19 patients. Lymphopenia can cause general immunosuppression and promote cytokine storms; both play important roles in viral persistence, viral replication, multi-organ failure, and death [31].

### 2.2. NK Cells and Cytotoxic T Lymphocytes Play Role in the Defense and Recovery of COVID-19

In COVID-19 patients, the proportions of the NK- and CD8+ T cells expressing CD-107a, IFN-γ, and IL-2 are also reduced, confirming the exhaustion of cytotoxic lymphocytes [27]. In one of the first case reports researchers investigated the breadth and kinetics of immune responses during a non-severe case of COVID-19 [32]. This case report provided valuable insights as it paralleled immunological results and clinical conditions of a diseased but previously healthy 45-year-old female patient from Wuhan, China, who travelled to Melbourne, Australia. The patient did not experience respiratory failure or an acute respiratory distress syndrome, did not require supplemental oxygenation, and was discharged after one week of hospitalization, which indicates a non-severe but a symptomatic course of the disease. Clinical findings in the lungs were observed on day 0 of hospitalization and were resolved after 10 days. It is important to consider the time sequence of immune responses: first, the activity of stem cells increases, then that of antigen-presenting cells and minimally active NK cells is enhanced; when their activity decreases, cytotoxic T lymphocytes (CD8+ containing granzymes A and B and perforin) increase, the level of helper T lymphocytes (CD4+) is sustained, and antibodies increase, with maximum IgG and IgM levels on day 20. Consistency of the immune system and a sequence was similar to instruments in a large orchestra. When lymphocyte cytotoxic activity (CD8+) is induced, the course of the disease is mild, and clinical findings in the lungs disappear within a few days without treatment. In such cases, it is probably not necessary to stimulate immune responses, because initial lymphopenia was not so pronounced. Nevertheless, in elderly patients or in those who are suffering from pre-existing chronic diseases, immunomodulation can substantially increase the levels of NK cells, accelerate the resolution of symptoms, and can prevent potential worsening of the disease to conditions such as primary viral pneumonia, secondary bacterial pneumonia, or venous thromboembolism [32].

### 2.3. Bacillus Calmette-Guérin, Innate Immunity and COVID-19

During the current pandemic of COVID-19, the health and safety of health care workers are of particular concern owing to their frequent exposure and high risks of infection. In March 2020, a clinical study on health care workers commenced in the Netherlands to examine the effects of stimulating innate immunity. Using a randomized controlled trial, the investigators aimed to stimulate innate immunity through Bacillus Calmette-Guérin vaccination to improve the clinical course of COVID-19 infections in health care workers and thereby reduce absenteeism, need for hospitalization and intensive care admission, and death rates during the current COVID-19 pandemic [33]. Up to now no results from studies with BCG immunization were published.

### 2.4. Complications of COVID-19 Can Be Treated in Community

There are three typical complications of COVID-19. The most important is viral immunosuppression, which can cause sometimes leucopenia, but much more often it is lymphopenia which occurs in the majority of severe COVID-19 patients, and it is strongly associated with a high mortality rate. The second complication, which can also affect a significant number of patients is venous thromboembolism caused by viral replication in endothelium and by immunothrombosis [34]. The last complication, which usually occurs at the end of the first week of the disease is secondary bacterial pneumonia caused by viral replication in the airway and alveolar epithelial cells followed by bacterial infection. Venous thromboembolism, immunothrombosis [34] and secondary bacterial cases of pneumonia are significant complications of COVID-19 caused directly or indirectly by the replication of the SARS-CoV-2 virus and the related lymphopenia. 

Although antibiotics do not directly affect SARS-CoV-2, viral respiratory infections often result in bacterial pneumonia. Some patients may die from bacterial co-infection rather than the virus itself. Therefore, bacterial co-infection and secondary bacterial infection are considered critical risk factors for the severity and mortality rates of COVID-19 [35].

All of these complications can be treated in the community at any general practitioner practice. Viral immunosuppression and its result lymphopenia can be treated by early applied immunomodulatory drugs, which can mitigate the effect of immunosuppression. The treatment of venous thromboembolism can start with acetylsalicylic acid or low–molecular-weight heparins [34]. The secondary bacterial pneumonia can be empirically treated by clavulanate-potentiated penicillin or macrolides.

When a patient is hospitalized for COVID-19, also corticosteroids are in the focus of the treatment. Dexamethasone is the first known steroid medicine that can save the lives of seriously ill patients, and it is shown in a randomized clinical trial by the United Kingdom that it reduced the death rate in COVID-19 patients [36].

## 3. Methods

The following databases were searched for clinical pharmacology and toxicology data: PubMed (https://pubmed.ncbi.nlm.nih.gov/ access date 15 August 2021), EBSCO (https://www.ebsco.com/ access date 15 August 2021), Scopus (https://www.scopus.com/ access date 15 August 2021), Cochrane Library (https://www.cochranelibrary.com/ access date 15 August 2021), WoS (https://www.webofscience.com/ access date 15 August 2021). The mesh words used during searching were: “inosine pranobex” or “isoprinosine” in combination with words “COVID-19” or “SARS-CoV-2” and “treatment” or “therapy” or “prevention” or “prophylaxis”. All retrieved studies are discussed in the present paper.

## 4. Inosine Pranobex

Since 1971 the synthetic compound inosine pranobex (IP), also known as inosine acedoben dimepranol or methisoprinol, has been licensed under brand names such as Isoprinosine for the treatment of viral infections in several countries worldwide. It consists of a combination of the p-acetamidobenzoate salt of N, N-dimethylamino-2-propanol, and inosine in a 3:1 molar ratio. It is an orally administered drug with pleiotropic effects known for its in vitro and in vivo immunopotentiation. Various mechanisms of action have been reported on humoral as well as the cell-mediated immune response. IP has been shown to help restore depressed cell-mediated responses of diverse etiologies that accompany a variety of clinical conditions [37,38,39,40,41]. It was shown that IP induces the Th1 cell-type response accompanied by elevated pro-inflammatory factors such as IL-2 and IFN-γ [42,43,44,45,46]. In addition, IP was reported to have direct antiviral effects [47,48,49]. It is used in the treatment of varicella, measles, a rare measles complication, i.e., subacute sclerosing panencephalitis (SSPE), infections caused by the herpes simplex virus (HSV), human papillomavirus (HPV), Epstein–Barr virus, cytomegalovirus, and viral influenza-like respiratory infections.

### 4.1. Clinical Trials with IP

A recent review paper provides a good overview of clinical trials conducted so far for the various indications such as treatment of HSV-1 and HSV-2, HPV, SSPV, Type B, and C viral hepatitis, Influenza and Rhinovirus infections, various immunodeficiency conditions, but also non-communicable diseases such as autoimmune diseases and chronic fatigue syndrome [50]. There is broad evidence supporting the use of IP in the treatment of genital and labial herpes simplex [51,52,53,54,55,56,57] and SSPE [58,59,60,61,62,63,64,65,66,67]. Weak to moderate, but still significant, effects were also reported against influenza [68,69,70]. The use of IP in immunocompromised patients in combating accompanying microbial infections is well established and supported by evidence and is well justified [71,72,73].

Acute respiratory infections are globally the most frequent type of viral infection. Severe forms are responsible for approximately 3.9 million deaths per year and are one of the leading causes of morbidity and mortality worldwide [74]. This number increased considerably during the spread of COVID-19. These infections are categorized as either upper or lower respiratory infections and are frequently caused by well-known viral pathogens including but not limited to influenza virus (types A and B), parainfluenza virus, respiratory syncytial virus, metapneumovirus (types A and B), coronavirus, rhinovirus, enterovirus, reovirus, bocavirus, and adenovirus.

Because of the current COVID-19 pandemic, it is important to consider the results of a study using clinical subjects with laboratory-confirmed acute viral respiratory infections that were conducted to compare the efficacy and safety of IP and a placebo drug [69]. In this study, the primary efficacy endpoint was considered the time of resolution of all influenza-like disease-associated symptoms.

In the study, 231 patients with influenza-like illness were treated with IP and compared with 232 patients receiving placebo in a clinical randomized, double-blind, multicenter study in the Czech Republic and Slovakia [69]. The study showed that the difference in time to resolution of all influenza-like symptoms between treatment groups was not statistically significant but showed faster improvement in subjects in the inosine pranobex group versus those in the placebo group (HR = 1.175; 95% CI: 0.806–1.714; *p* = 0.324). A subgroup analysis in patients <50 years of age revealed a statistically shorter time to resolution of symptoms in those without related ongoing disease and those with BMI < 30 kg/m^2^. Safety was evaluated through analysis of adverse events, vital signs, and physical examinations. The study showed that IP is a generally safe drug for the treatment of patients with respiratory infections.

### 4.2. Mechanism of Action in the Treatment of COVID-19 with IP

IP has been used due to its immunomodulatory and anti-viral properties for decades. Several mechanisms of action have been proposed. A review paper by Sliva et al. provides a comprehensive overview [50]. Beyond the review, we want to emphasize two recently described mechanisms that, we believe, could be successfully exploited in the early treatment of COVID-19.

#### 4.2.1. Natural Killer Cell Early Response

Natural killer (NK) cells are effector cells of the innate immune system that control human infection in its early stage. They are a separate lymphocyte lineage, with cytotoxicity as well as cytokine-producing effector function [75,76,77]. The complex mechanism and role of NK cells in protection from various classes of viruses have been reasonably established [77,78,79,80,81,82]. NK cells have a substantial importance in preventing several types of tumors and infectious pathogens from causing widespread tissue destruction and becoming disseminated in the organism. NK cell modulation, therefore, has therapeutic potential for early impact across a variety of viral pathogens.

A clinical trial with 27 individuals followed the effect of IP administration (14 days) on multiple lymphocyte subsets–CD19 + B cells, CD3 + T cells, CD4 + T-helper cells, FoxP3hi/CD25hi/CD127lo regulatory T cells (Tregs), CD3 −/CD56 + NK cells, and CD3 +/CD56 + NKT cells, together with serum immunoglobulins and IgG subclasses. As early as within 1.5 h of receiving the drug, one-half of the cohort had a durable (>5 days) rise in NK cells. By the fifth day, all but one of the subjects had higher proportions of NK cells, and these elevations—effectively two-fold or higher—were maintained until the end of the study [82]. The induced NK population was not deficient in Granzyme A and Perforin; hence, their clearing function in the organism was reinforced by the elevated number of NK cells.

Activated NK cells can recognize virus-infected cells and kill them using perforin and granzymes. Thus, from the beginning, natural immunity is significantly involved in the destruction of infected cells and fundamentally affects the prognosis of viral infections. NK cells are capable of eliminating virus-infected cells rapidly and directly, without antigen presentation or recognition. Stimulated by infections, cytokines, stress, or other cells of the immune system, NK cells release perforin and granzyme that kills infected cells [80]. NK cells also regulate immune responses employing cytokine secretion, and, further, they can couple with apoptosis-inducing receptors on target cells and trigger apoptosis [81,82,83]. The rather recent finding of IP boosting phenotypically competent NK responses reinforces the drug’s benefit in treating conditions associated with viral infections and could potentially be exploited in the early treatment of COVID-19 [82]. NK cells play also a very important role at the interface between innate and adaptive immunity in defense of viral infection. Nevertheless, the key role lies in the NK cell cytotoxicity [84,85].

#### 4.2.2. Induced NKG2D Ligand Expression

IP is a potent medication for treating or preventing viral infections under various conditions, including patients who are elderly but otherwise healthy. This is particularly important since, in these populations, NK performance is known to be compromised, and insufficient performance may be a key contributor to high rates of viral infections associated with immunosenescence [86,87]. Activating and inhibitory receptors on the surface of NK cells are responsible for recognizing ligands on target cells, and the relative degree of expression of these receptors and the associated response of the linked signal cascades determine the level of NK cell activation and cytotoxicity [88]. Studies on IP demonstrated that its immunomodulatory activity is characterized by enhancing lymphocyte proliferation, cytokine production, and NK cell cytotoxicity [88].

Another proposed pharmacodynamic activity is based on the ability of IP to alter cellular immunity through the induction of NKG2D ligand expression on target cells, thereby enhancing immune cell activation through the NKG2D receptor. This mechanism seems to be additive to the above-described NK activation since it acts inside infected cells and makes them more visible to T lymphocytes. Using both targeted metabolic interventions and unbiased metabolomic studies, it was demonstrated that IP causes an increase in the intracellular concentrations of purine nucleotides and tricarboxylic acid (TCA) cycle intermediates and leads to NKG2D ligand induction. The degree of NKG2D ligand induction was functionally significant, leading to increased NKG2D-dependent target cell immunogenicity. These findings demonstrate that the immunomodulatory properties of IP are also due to metabolic activation with NKG2D ligand induction [88].

In addition to venous thromboembolic disease or immunothrombosis and secondary pneumonia, the underlying problem of COVID-19 is initial immunosuppression caused by the virus. This manifests as selective lymphopenia, which, however, can be prevented by the use of IP. Due to the above-mentioned decrease of immune system activities by the SARS-CoV-2 virus, IP can not only prevent viral immunosuppression but also restore the immune system to its original state rather quickly.

The results of these two studies indicate that IP can boost the levels of phenotypically competent NK cells in healthy individuals during conditions associated with acute viral respiratory infections, and it can be used to improve potentially compromised immune functions [82,88].

IP is not a causal drug but a drug that can help overcome the initial key complications of COVID-19 infections and can thus substantially alter the course of the disease.

## 5. Use of IP in COVID-19 Patients

There have been two ongoing multicenter randomized clinical trials with IP in patients with moderate COVID-19 in India since the summer of 2020. The first was an open-label proof of concept study with 60 patients. It focused on the effect of IP in COVID-19 patients when used along with the standard of care vs. the standard of care. The study was registered on 14 August 2020 (http://www.ctri.nic.in/Clinicaltrials/pdf_generate.php?trialid=46240&EncHid=&modid=&compid=%27,%2746240det%27 access date 4 November 2021). In this study sub-group analysis of patients showed that inosine pranobex, when added to standard of care containing (CSC) Azithromycin and Hydroxychloroquine with or without Ivermectin, produced significantly higher clinical response (CR) at Day-14 than the only standard of care (100.00% vs. 69.23%; *p* = 0.03). Overall, there was a trend of (numerically) higher CR, at Day 7, 14 and 21 in the IP + CSC group compared to the CSC group; however, statistical significance was not be reached. This may be because of the small sample size of the study variability in the current standard of care among the different sites. IP was well tolerated. The first study was followed by a phase 3, double-blind, placebo-controlled randomized trial studying the effects of adding IP to the standard of care in COVID-19 patients (http://www.ctri.nic.in/Clinicaltrials/pdf_generate.php?trialid=49972&EncHid=&modid=&compid=%27,%2749972det%27 access date 4 November 2021). So far, no randomized controlled clinical trial to study IP in patients with COVID-19 has been published.

Between June and October 2020, IP was used by doctors in several retirement and nursing homes in the Czech Republic since there were no medication treatments available at that time. The decision to treat SARS-CoV-2 PCR positive (PCR+) patients was made based on the above described previously published study indicating that IP could be effective and safe in treating acute viral respiratory infections [69].

**Figure 1:**
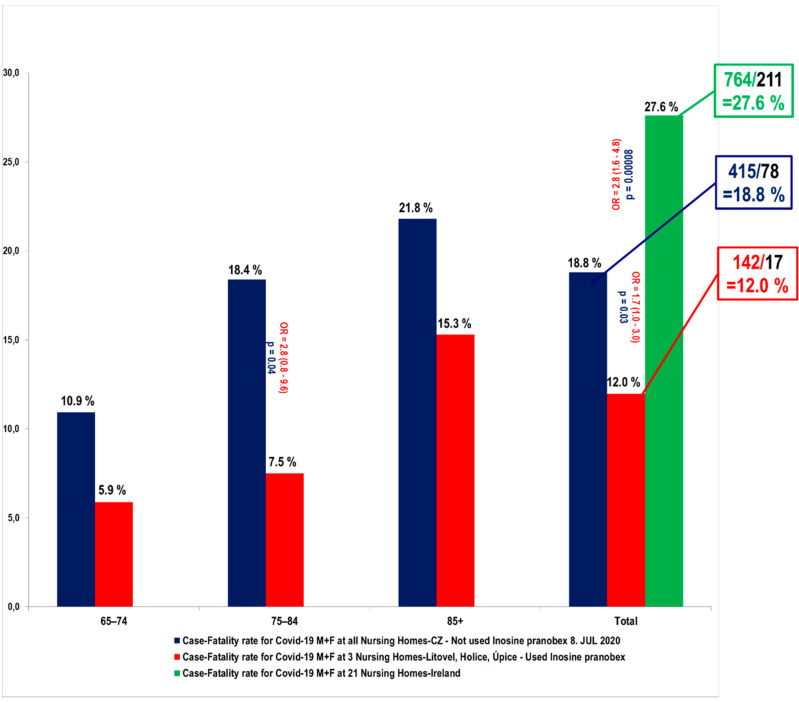
Comparison of the case fatality rate (CFR) for COVID-19 of the residents of three nursing homes (142/17) to the CFR from all nursing homes in the Czech Republic (415/78) and to the CFR of 21 NH in Ireland (764/211).

The most recent study from 2020 was conducted as a retrospective analysis of treatment without protocols, which reduces its scientific value; however, it still created an opportunity to study the real-world effects of IP in COVID-19 patients [89]. The retrospective analysis compared the treated group (NPCR+ = 142 patients, of which 17 died) defined across the three nursing homes. Residents from all nursing homes in the Czech Republic were selected as the first control group until 8 July 2020, since IP presented no treatment option for nursing homes until that time. The control group consisted of 415 PCR+ residents, of which 78 died (CFR 18.8%). Notable is the case fatality rate of 12% among those in the above-mentioned IP-treated patients in Czech nursing homes as compared with the case fatality rate of 27.6% (PCR+ = 764/211 patients died) reported in Irish nursing homes (Figure 1) [90]. Case fatality rate calculated as a percentage of those who died from those PCR+ was reported as the sole outcome, which further limits the analyses.

The study reported a notable difference in the COVID-19 case fatality rate between those treated with IP (12%) and those without IP treatment (28.6%). These figures were obtained from a rather heterogeneous set of patients and could hence be affected by a confounding effect, such as the underlying health condition or age, to some degree. In perhaps the most comparable set of patient data from only one of the homes (in the town of Litovel, NPCR+ = 33), the case fatality rate observed among those treated with IP (NIP+ PCR+ = 19) compared with those untreated (NIP− PCR+ = 14) was 5.3% versus 28.6%, respectively. This study suggests that further clinical studies should be conducted to provide evidence of IP’s efficacy in COVID-19 patients.

A randomized controlled study took place in Ecuador between March and April 2020 [91]. In total, 60 symptomatic COVID-19 patients were evenly and randomly divided so that 30 were treated with IP (methisoprinol) along with other COVID-19 medications, and 30 received only standard medications. The efficacy endpoints after 15 days of therapy were the presence of clinical signs, PCR positivity, oxygen saturation (SO2 > 90%), and axial lung tomography (with or without lessons). Patients treated with IP presented demonstrably better outcomes in all monitored endpoints (Table 1).

## 6. Conclusions

IP has long been known for its beneficial effect in the treatment of many viral infections, including influenza and other influenza-like states. There are plausible immunomodulatory mechanisms for its actions, which promise broad therapeutic effects across a variety of viral pathogen variants. IP alone will most likely not lead to a full recovery, but early immunomodulatory intervention could alter the course of the disease.

Clinical and immunological studies and analyses conducted over the past six years have demonstrated the effect of IP, via natural killer cells and cytotoxicity, for treating the majority of investigated viral infections; this efficiency will hopefully be transferrable to the currently spreading acute viral respiratory infection COVID-19.

It seems that early application of IP can mitigate initial viral immunosuppression and initial lymphopenia, which are strongly associated with COVID-19 progression, hospitalization, and mortality. We can conclude that to date limited clinical evidence supporting IP efficacy exists, but a retrospective study, as well as a small local study from Ecuador, or India have provided a distinct signal that IP could be beneficial in the treatment of COVID-19 patients.

Since this drug was in use in the Czech Republic and potentially elsewhere, ananalysis of the health reimbursement data in those treated with IP combined with their subsequent health care utilization and mortality data could provide valuable insight into the real-world effectiveness of IP in COVID-19 patients and thus complement ongoing clinical studies.

## Figures and Tables

**Table 1:** Endpoints in a clinical study by Borges et al., 2020 [91].

Indicator	Results after >15 Days of Therapy	The Experimental Group (with Methisoprinol)		Control Group (without Methisoprinol)		Statistically Significant Risk Ratio ≤ 0.57
Clinical Picture	With Clinical Signs	3/30	(10%)	20/30	(66.6%)	0.14
Oxygen Saturation	SO2 > 90%	30/30	(100%)	23/30	(76.6%)	0.0
PCR Test	PCR Negative	28/30	(93.3%)	20/30	(66.6%)	0.20
Axial Tomography	No Lung Lesions	29/30	(96.6%)	4/30	(13.3%)	0.038

Note: The data obtained were analyzed and presented as frequency (F) and percentage (%); the correlation was established using relative risk, considering statistically significant differences when the RR value is equal to or less than 0.57, with the results expressed in Table 1 [84].

## Data Availability

All the data can be found in cited publications.
